# Protective Effect of Adipose-Derived Mesenchymal Stem Cell Secretome against Hepatocyte Apoptosis Induced by Liver Ischemia-Reperfusion with Partial Hepatectomy Injury

**DOI:** 10.1155/2021/9969372

**Published:** 2021-08-18

**Authors:** Zhihui Jiao, Yajun Ma, Yue Wang, Tao Liu, Qianzhen Zhang, Xiaoning Liu, Chenxi Piao, Boyang Liu, Hongbin Wang

**Affiliations:** ^1^College of Veterinary Medicine, Northeast Agricultural University, Harbin, China; ^2^College of Wildlife and Protected Area, Northeast Forestry University, Harbin, China

## Abstract

Ischemia-reperfusion injury (IRI) is an inevitable complication of liver surgery and liver transplantation. Hepatocyte apoptosis plays a significant role in the pathological process of hepatic IRI. Adipose-derived stem cells (ADSCs) are known to repair and regenerate damaged tissues by producing bioactive factors, including cytokines, exosomes, and extracellular matrix components, which collectively form the secretome of these cells. The aim of this study was to assess the protective effects of the ADSCs secretome after liver ischemia-reperfusion combined with partial hepatectomy in miniature pigs. We successfully established laparoscopic liver ischemia-reperfusion with partial hepatectomy in miniature pigs and injected saline, DMEM, ADSC-secretome, and ADSCs directly into the liver parenchyma immediately afterwards. Both ADSCs and the ADSC-secretome improved the IR-induced ultrastructural changes in hepatocytes and significantly decreased the proportion of TUNEL-positive apoptotic cells along with caspase activity. Consistent with this, P53, Bax, Fas, and Fasl mRNA and protein levels were markedly decreased, while Bcl-2 was significantly increased in the animals treated with ADSCs and ADSC-secretome. Our findings indicate that ADSCs exert therapeutic effects in a paracrine manner through their secretome, which can be a viable alternative to cell-based regenerative therapies.

## 1. Introduction

Hepatic ischemia-reperfusion injury (HIRI), a precursor to liver dysfunction and liver failure [1], is an inevitable complication of shock, trauma, hepatectomy, liver transplantation, and other surgical procedures [2–4]. The pathophysiological process of HIRI involves excessive production of reactive oxygen species (ROS), activation of Kupffer cells and other inflammatory cells, and calcium overload, which eventually lead to hepatocellular apoptosis [5]. Although orthotopic liver transplantation (OLT) is an effective treatment for terminal liver dysfunction, it is limited by organ shortage, high costs, immune rejection, and transplant-related complications [6]. HIRI is still an unresolved clinical issue, and an effective strategy is urgently needed to alleviate HIRI and improve patient prognosis.

Stem cell therapy is a promising approach for tissue repair and regeneration [7–9]. Mesenchymal stem cells (MSCs) in particular have shown encouraging results against inflammatory, degenerative, and ischemia-reperfusion diseases [10–12] since they can be isolated from multiple sources, including adipose tissue [13], bone marrow, dental pulp [14], umbilical cord blood [15], tonsils [16], oral cavity [17], and amniotic fluid [18]. Adipose-derived stem cells (ADSCs) are increasingly being considered a promising tool for cellular therapy and tissue engineering [19]. Depending on the environmental stimuli, ADSCs can differentiate into osteoblasts, adipocytes, and hepatocytes [20] and are therefore highly suitable for cell-based therapy in multiple organ systems. However, the clinical application of stem cells is limited by long-term safety concerns, such as unwanted differentiation [21], potential tumorigenicity [22], and elimination by the receptor immune system [23]. Therefore, stem cell transplantation is still at the experimental stage [24].

The regenerative effects of transplanted stem cells are mainly attributed to the paracrine regulation of endogenous cells via secreted factors [25]. The secretome of a cell population refers to the biologically active factors secreted by the cells into the extracellular space, including soluble proteins, free nucleic acids, lipids, and extracellular vesicles [26], that aid in intercellular communication and transport. Several studies have demonstrated the regenerative potential of the stem cell secretome, which can obviate some of the pressing concerns of cell-based therapies, including immune rejection, tumorigenicity, and emboli formation.

Adipose-derived stem cell conditioned medium (ADSC-CM) or secretome has shown remarkable therapeutic effects in small-animal models of angiogenesis [27], diabetic pain [28], wound healing [29], glucose metabolism [30], etc. While stem cell therapy has been investigated in animal models of partial hepatectomy, little is known regarding the effect of ADSC-secretome on HIRI in large animals. In this study, we established laparoscopic hepatic ischemia-reperfusion and partial hepatectomy in miniature pigs and transplanted ADSCs or the ADSC-secretome directly into the liver parenchyma. The ADSC-secretome alleviated apoptosis in the hepatocytes and improved cellular ultrastructure. Our findings show that the ADSC-secretome is a safe and effective strategy against HIRI.

## 2. Materials and Methods

### 2.1. ADSC Culture and Preparation of Conditioned Medium (CM)

Adipose tissues were obtained from the subcutaneous abdominal fat and digested with collagenase I at 37°C for 45 min with continuous shaking. After neutralizing enzyme activity with L-DMEM (low glucose-Dulbecco's modified Eagle medium) supplemented with 10% FBS (Clark, USA), the homogenate was filtered and centrifuged, and the ADSCs were suspended in L-DMEM supplemented with 10% FBS, 2 mM L-glutamine, and 100 *μ*g/ml penicillin and streptomycin (Solarbio, China). The cells were cultured at 37°C under 5% CO_2_ in a humidified incubator (Galaxy 170 S, Eppendorf, Germany). The ADSCs were characterized as previously described [31], cultured in serum-free L-DMEM for 48 h. Then, the medium was aspirated and centrifuged at 1000 g for 15 min at 4°C to remove the cell debris. The supernatant was then centrifuged at 5000 g for 50 min at 4°C using 3 kDa MWCO (Millipore, Billerica, USA) to concentrate it by 25-fold. The CM aliquots were transferred to sterile 1.5 ml EP tubes and stored at -80°C.

### 2.2. Surgical Procedure

Twenty-four miniature pigs (age: 4-6 months, body weight: 20-25 kg) were provided by the Miniature Pig Farm of the College of Life Sciences (Harbin, China). The animals were housed at 20°C under a 12 h light-dark cycle and fed piglet diet (Shenzhen Jinxinnong Feed, China) and tap water ad libitum. The animals were randomly divided into the (untreated) IRI, DMEM control, CM, and ADSC groups (*n* = 6 per group). After 12 h fasting and 2 h water deprivation, the animals were anesthetized with isoflurane inhalation and subjected to laparoscopic left hepatectomy after right hepatic ischemia for 60 min. Immediately after the operation, the liver parenchyma was injected with saline (IRI group), DMEM, ADSCs (1 × 10^6^ P4 cells/kg body weight) or ADSC-secretome (CM equivalent to 1 × 10^6^ P4 ADSCs/kg). The animals were euthanized by injecting 4% tolfedine (Vetoquinol S.A., France). Liver tissues were laparoscopically harvested preoperation and 1, 3, and 7 days postoperation. All the animals survived during the entire duration of the experiment due to the minimally invasive surgery.

### 2.3. Transmission Electron Microscopy

The liver samples were cut into 1 mm^3^ pieces and fixed with 2.5% glutaraldehyde. After routine dehydration, the tissues were embedded, sectioned, and stained with lead citrate and uranyl acetate. The ultrastructural changes in the hepatocytes were observed using an H-7650 transmission electron microscope (Hitachi, Japan).

### 2.4. TUNEL Analysis

The liver tissues were fixed with 4% paraformaldehyde (*n* = 6 per group), embedded with paraffin, and cut into sections. After dewaxing and dehydrating, the sections were stained with TUNEL assay using an *In Situ* Cell Death Detection Kit (Roche, Germany) according to the manufacturer's instructions. The TUNEL-positive cells were counted in five random fields of each section, and its percentage relative to the total number of hepatocytes was calculated.

### 2.5. Caspase Activity Analysis

Liver tissues were homogenized with lysis buffer (*n* = 6 per group), and the activities of caspase 3, caspase 8, and caspase 9 were determined using specific Caspase Activity Assay Kits (Solarbio, China) according to the manufacturer's instructions.

### 2.6. Real-Time Quantitative PCR Analysis

Total RNA was extracted from the liver tissues using a TRIzol reagent (Invitrogen, Shanghai, China) according to the manufacturer's instructions. Reverse transcription was performed using a PrimeScript™ RT Reagent Kit (Takara, Japan). RT-qPCR was performed using the SYBR Green Kit in a LightCycler 480 System (Roche Applied Science, Penzberg, Germany) with the following cycling parameters: predenaturation at 95°C for 30 s, followed by 40 cycles of denaturation at 95°C for 5 s, and annealing and elongation at 60°C for 1 min. The threshold cycle (CT) values were calculated by the 2^−∆∆Ct^ method (Livak and Schmittgen, 2001). The primers are listed in Table 1.

### 2.7. Western Blotting

Liver tissues were homogenized using a Tissue Protein Extraction Reagent (Beyotime, Shanghai, China) for 30 min at 4°C. The homogenates were centrifuged at 12,000 g for 15 min at 4°C, and the protein concentration was determined using a Bicinchoninic Acid (BCA) Protein Assay Kit (Beyotime, Shanghai, China). Equal amounts of protein were separated by sodium dodecyl sulfate-polyacrylamide gel electrophoresis (SDS-PAGE) and then transferred onto nitrocellulose (NC) membranes. After blocking with 5% nonfat milk in TBST for 2 h at room temperature, the membranes were incubated overnight with anti-P53, anti-Bcl-2 (Wanlei Biology, Shenyang, China), anti-Bax, and anti-*β*-actin (Sangon Biotech, Shanghai, China) primary antibodies. The membranes were then washed with TBST and incubated with horseradish peroxidase-conjugated secondary antibody (ImmunoWay, Plano, USA) at room temperature for 2 h. Following another wash with TBST, the blots were developed using a Meilunbio® fg super-sensitive ECL luminescence reagent (Meilunbio, Dalian, China) and imaged using the Tanon 5200 Imaging System (Tanon Science & Technology Co., Shanghai, China). The relative density of the target bands was quantified using ImageJ software.

### 2.8. Immunohistochemistry

Paraffin-embedded tissue sections were deparaffinized, dehydrated, and treated with 3% hydrogen peroxide in the dark for 10 min to inactivate the endogenous peroxidases. After heating in 0.01 M citrate buffer in the microwave for 10 min for antigen retrieval, the sections were cooled to room temperature, blocked with bovine serum albumin (BSA), and incubated overnight with anti-Fas and anti-Fasl primary antibodies (ImmunoWay, Plano, USA) at 4°C. The sections were then incubated with a streptavidin-labeled HRP secondary antibody (ZSGB, Beijing, China) for 30 min at room temperature, followed by a DAB solution for 3 min. After counterstaining with hematoxylin, the positively stained sections were quantified with the Image-Pro Plus 6.0 software (Media Cybernetics, USA). Six tissue sections per group and five random fields per slide were analyzed.

### 2.9. Statistical Analysis

All data were analyzed with GraphPad Prism 7.0 (GraphPad Software, USA) and expressed as mean ± SD. One-way ANOVA was used to compare different groups, and *P* < 0.05 was considered statistically significant.

## 3. Results

### 3.1. ADSCs/ADSC-Secretome Relieved the Ultrastructural Damage in Injured Hepatocytes

**Figure 1:**
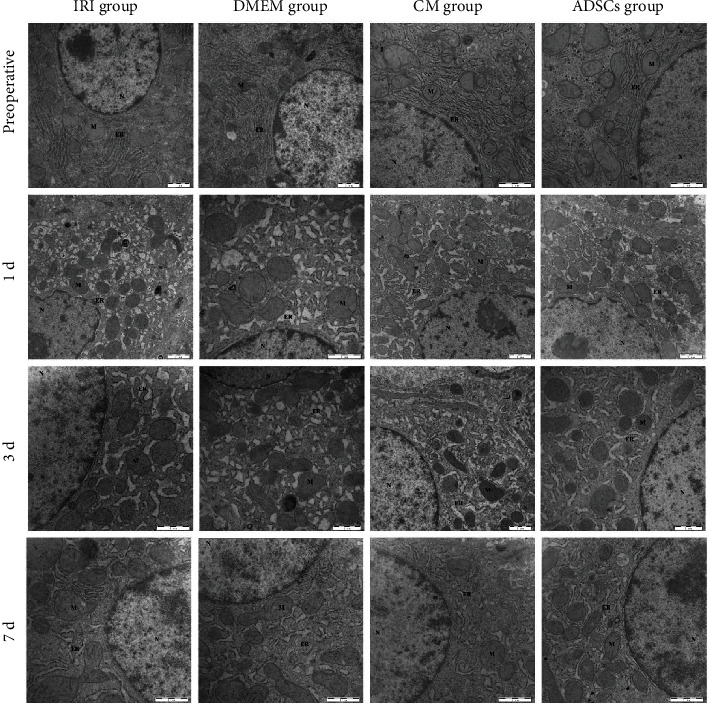
The ultrastructural changes in hepatocytes.

As shown in the electron micrographs in Figure 1, the nuclei, mitochondria, and endoplasmic reticulum (ER) of the preoperative hepatocytes were normal. Within a day after HIRI, however, significant ultrastructural changes were observed, such as nuclear membrane shrinkage, chromatin condensation at the edges, mitochondrial swelling, and severe ER expansion. These changes were less evident 3 days postoperation and largely subsided by day 7 with only a slight expansion of the ER. Transplantation of either ADSCs or the ADSC-secretome significantly alleviated mitochondrial swelling and ER expansion postoperation, whereas DMEM had no effect. The results indicate that ADSCs/ADSC-secretome can improve the ultrastructural changes in hepatocytes after ischemia-reperfusion and partial hepatectomy.

### 3.2. ADSCs/ADSC-Secretome Decreased Postischemic Hepatocyte Apoptosis

**Figure 2:**
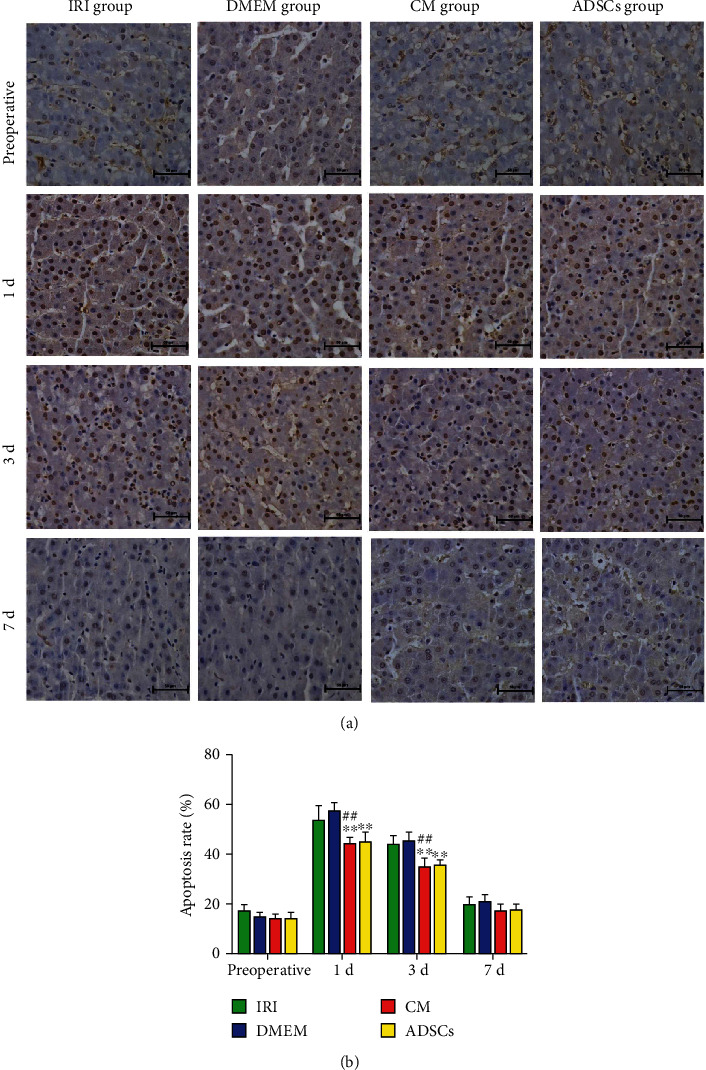
Hepatocyte apoptosis rates: (a) representative images of TUNEL-stained liver tissues from the indicated groups and at the indicated time points (magnification 400x); (b) apoptosis rate of hepatocytes. ^∗∗^*P* < 0.01 compared to IRI group and ^##^*P* < 0.01 compared to DMEM group.

As shown in Figure 2(a), numerous TUNEL-positive apoptotic cells were present in the hepatic tissues 1 and 3 days after surgery. The apoptosis rates at both time points were significantly higher in the untreated and DMEM control animals compared to those transplanted with ADSCs and the ADSC-secretome (Figure 2(b); *P* < 0.01). Thus, the ADSCs and their secretome can alleviate IRI-induced apoptosis in the hepatocytes.

### 3.3. ADSCs/ADSC-Secretome Decreased Caspase Activity in Hepatocytes

**Figure 3:**
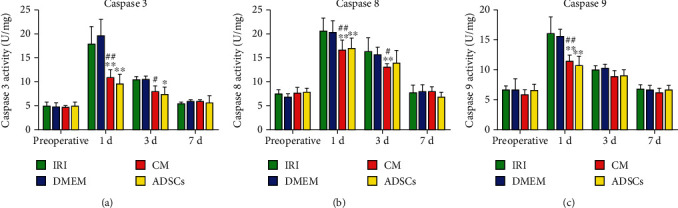
Caspase activity levels: (a) caspase 3, (b) caspase 8, and (c) caspase 9. ^∗^*P* < 0.05 and ^∗∗^*P* < 0.01 compared to IRI group; ^#^*P* < 0.05 and ^##^*P* < 0.01 compared to DMEM group.

To further elucidate the mechanistic basis of the antiapoptotic effects of ADSCs/ADSC-secretome, we analyzed the activity of multiple caspases in the liver tissues after ischemia-reperfusion and partial hepatectomy. As shown in Figure 3, the activity of caspase 3, caspase 8, and caspase 9 peaked 1 day after surgery but was significantly reduced in the ADSCs/CM-treated groups (*P* < 0.01). In addition, caspase 8 activity remained significantly higher on day 3 postoperation in the IRI and DMEM control groups compared to the CM-treated group (*P* < 0.01, *P* < 0.05). Caspase 3 activity levels dropped by day 3 even in the untreated groups, although the reduction was more significant in the CM-treated versus DMEM groups (*P* < 0.05). In contrast, caspase 9 activity was similar across all groups 3 days after operation. Caspase activity levels were restored to normal in the untreated animals at day 7 postoperation.

### 3.4. ADSCs/ADSC-Secretome Altered the Expression of Apoptosis-Related Factors

**Figure 4:**
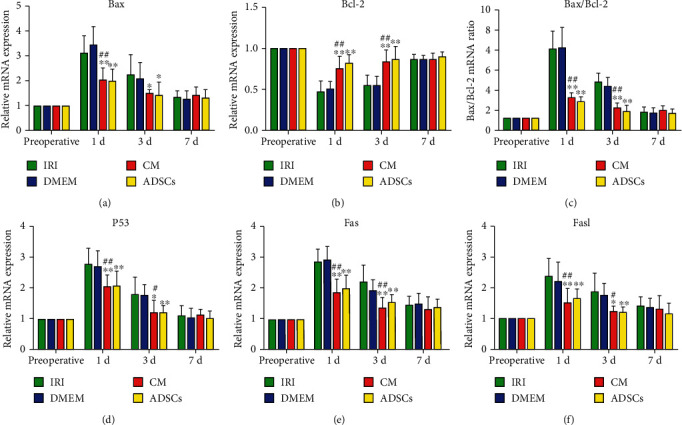
Expression levels of apoptosis-related genes in liver tissue. ^∗^*P* < 0.05 and ^∗∗^*P* < 0.01 compared to IRI group; ^#^*P* < 0.05 and ^##^*P* < 0.01 compared to DMEM group.

**Figure 5:**
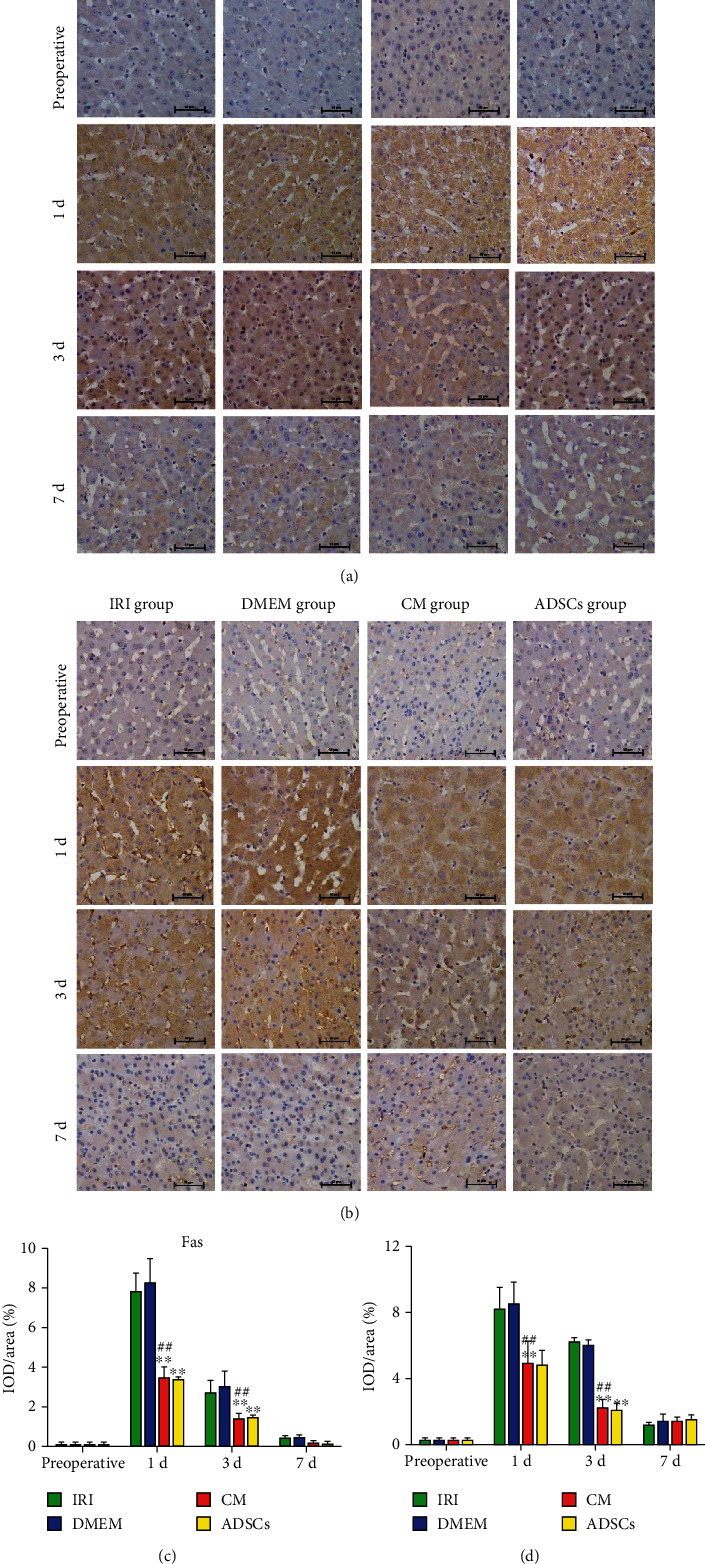
In situ expression levels of Fas and Fasl protein in liver tissues. (a, b) Representative images of immunohistochemically stained Fas and Fasl protein in the indicated groups (magnification 400x). (c, d) Relative expression levels of Fas and Fasl. ^∗^*P* < 0.05 and ^∗∗^*P* < 0.01 compared to IRI group; ^#^*P* < 0.05 and ^##^*P* < 0.01 compared to DMEM group.

The antiapoptotic effects of the ADSCs/ADSC-secretome were further confirmed by analyzing the expression levels of apoptosis-related proteins including Bax, Bcl-2, P53, Fas, and Fasl. As shown in Figure 4(a), Bax mRNA levels increased significantly after surgery and were downregulated by both ADSCs and the ADSC-secretome on day 1 (*P* < 0.01) and day 3 (*P* < 0.05) postoperation. On the other hand, the antiapoptotic Bcl-2 was downregulated after surgery and increased in the animals treated with ADSCs/ADSC-secretome on days 1 and 3 postoperation (*P* < 0.01 compared to the DMEM group; Figure 4(b)). Consistent with this, the ADSCs and ADSC-secretome significantly decreased the Bax/Bcl-2 ratio at both time points (*P* < 0.01, Figure 4(c)). The upstream regulator P53 was also downregulated by the ADSC-secretome and ADSCs on days 1 (*P* < 0.01 for both) and 3 (*P* < 0.05 and *P* < 0.01) compared to the untreated IRI and DMEM groups (Figure 4(d)). The Fas and Fasl transcripts also showed similar trends (*P* < 0.01 for all; Figures 4(e) and 4(f)). Taken together, the ADSCs and ADSC-secretome upregulated the antiapoptotic genes and suppressed the proapoptotic genes in hepatocytes. Consistent with the above, cytoplasmic expression of Fas and Fasl proteins increased in the liver parenchyma of all groups after surgery (Figures 5(a) and 5(b)). However, both ADSCs and the ADSC-secretome significantly reduced the *in situ* expression of both on days 1 and 3 after surgery (*P* < 0.01 and *P* < 0.05, respectively, as indicated in Figures 5(c) and 5(d)).

**Figure 6:**
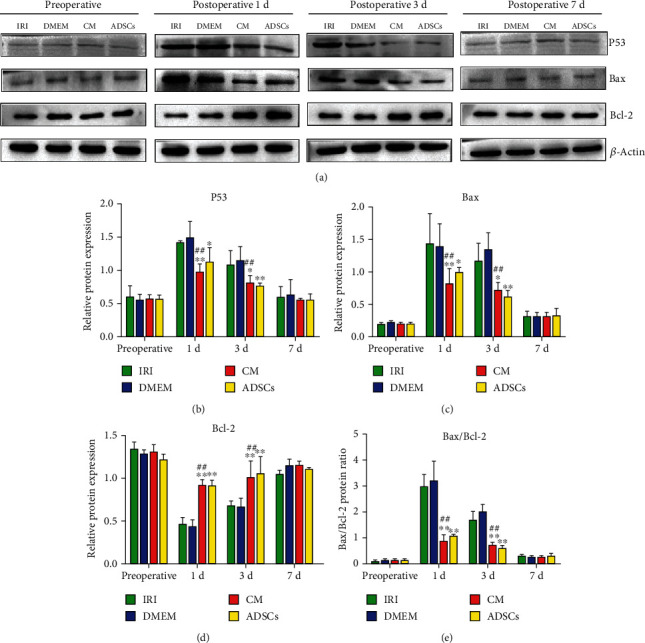
Expression levels of apoptosis-related proteins in liver tissue: (a) representative immunoblot showing P53, Bax, Bcl-2, and *β*-actin levels in the indicated groups. (b–e) Quantification of P53, Bax, Bcl-2, and Bax/Bcl-2 ratio. ^∗^*P* < 0.05 and ^∗∗^*P* < 0.01 compared to IRI group; ^#^*P* < 0.05 and ^##^*P* < 0.01 compared to DMEM group.

Likewise, the ADSCs and the ADSC-secretome also significantly reduced P53 and Bax protein expression on days 1 (*P* < 0.01 and *P* < 0.05, respectively) and 3 (*P* < 0.05 and *P* < 0.01, respectively) after surgery (Figures 6(a) – 6(c)) and upregulated Bcl-2 at both time points (*P* < 0.01; Figures 6(a) and 6(d)). Consistent with this, the Bax/Bcl-2 ratio was significantly lower in the ADSC/CM-treated animals compared to the untreated IRI and DMEM control on days 1 and 3 (*P* < 0.01) postoperation (Figure 6(e)).

## 4. Discussion

Laparoscopic hepatectomy has been successfully used to establish liver injury in large animal models [32, 33]. Multiple studies show that the stem cell-derived secretome plays an active role in alleviating the symptoms of ischemia-reperfusion [34–36]. However, it is unclear whether the ADSC-secretome in particular exerts an active therapeutic effect on HIRI. Therefore, the aim of our study was to evaluate the effect of ADSCs and its secretome on hepatocyte apoptosis after HIRI combined with partial hepatectomy.

ADSCs are adult stem cells with immune regulation, secretion of growth factors, promotion of blood vessel formation, and tissue regeneration. Compared with the secretome from other stem cells, the ADSC-secretome has obvious advantages, including no bioethical restrictions of embryonic stem cells, large-scale production, easy storage and transportation, and fast therapeutic effect. So, it is economical and practical in clinical practice and brings hope to the application of cell-free therapy. The miniature pig is a kind of experimental animal with abundant adipose tissue. In addition, miniature pigs are suitable experimental animals for studying pathological changes in organs due to the anatomical and physiological similarities with humans. ADSCs from miniature pigs secrete proteins such as ANG-1, ANG-2, VEGF, and b-FGF [37]. Previous studies have demonstrated that ANG-1 can promote the expression of the Bcl-2 protein [38]. b-FGF can participate in the process of cellular mitosis and induce cell proliferation and differentiation which prevent cell apoptosis [39]. Therefore, the secreted protein may have a role in the antiapoptotic effect of the ADSC-secretome. TUNEL staining is a shared method to detect cell apoptosis. After HIRI, TUNEL staining showed an increase in the number of apoptotic liver cells [40]. Yi-Xing et al. found that MSC-CM has a direct inhibitory effect on sinusoidal endothelial cell apoptosis by TUNEL staining [41]. And the CM of human umbilical cord mesenchymal stem cells can also reduce the percentage of TUNEL-positive cells to play a protective effect on the cells [42]. Similarly, our results show that the ADSC-secretome from miniature pigs reduced the numbers of apoptosis cells by TUNEL staining after HIRI combined with partial hepatectomy.

HIRI is a complex pathophysiological process that involves ischemia, hypoxia, early reperfusion, and reperfusion injury. Liver tissue reperfusion generates a large amount of ROS, and the resulting oxidative stress accelerates tissue inflammation and cell death [43]. In addition, the Ca^2+^ overload during ischemia-reperfusion alters mitochondrial membrane permeability, which lowers ATP production and oxygen consumption, thereby affecting the survival of liver cells. Apoptosis was first described by Kerr [44] in hepatocytes. We detected a significant increase in apoptotic cells in the liver tissue after ischemia-reperfusion, which correlated to ultrastructural changes such as chromatin disintegration, mitochondrial swelling, and endoplasmic reticulum expansion. Although antioxidants improve the symptoms of HIRI, they are not feasible for clinical application [45]. MSC-CM protects cells from apoptosis [42, 46] and can improve mitochondrial function and reduce hepatocyte apoptosis in nonalcoholic fatty liver disease [47]. In addition, previous studies have demonstrated antioxidative and anti-inflammatory effects of ADSCs [11, 37]. Consistent with this, both ADSCs and the ADSC-secretome alleviated apoptosis following HIRI, as indicated by improved organelle structure, lower levels of caspases, and downregulation of proapoptotic genes and proteins.

Hepatocyte apoptosis is involved in maintaining the normal physiological functions of the liver and plays an important role in acute or chronic diseases of the liver, such as I/R injury, viral hepatitis, alcoholic and nonalcoholic liver diseases, and cholestatic diseases [48]. Therefore, understanding the mechanism of hepatocyte apoptosis is of great significance for the treatment of liver diseases. Apoptosis is mediated via the endogenous mitochondrial pathway, exogenous death receptor pathway, and endoplasmic reticulum pathway and regulated by the caspase family, Bcl-2, and P53 among others. The P53 protein forms a complex that transports the Bax protein to the nucleus, which promotes Bax expression and inhibits Bcl-2 to mediate early apoptosis [49]. Furthermore, various death signals depolarize the mitochondrial membrane by opening the transition pore, which releases cytochrome C into the cytoplasm. Cytochrome C forms multimers with Apaf1 and ATP/d ATP, which activate the caspase 9 precursor by promoting self-cleavage. Cleaved caspase 9 triggers the downstream caspase 3 and caspase 7 cascade, eventually culminating in apoptosis. A previous study showed that the CM of bone marrow mesenchymal stem cells (BMSCs) alleviated neuronal apoptosis by downregulating Bax and the cleaved caspase 3/caspase ratio and increasing Bcl-2 levels [50]. The Bcl-2 protein family is currently the most valuable protein family that regulates apoptosis. Antiapoptotic proteins Bcl-2, Bcl-x, and Bcl-w block the apoptotic cascade by inhibiting cytochrome C release [51]. Another study demonstrated that ADSC-CM can significantly reduce the expression of proapoptotic proteins such as Bax during ischemia-reperfusion-induced cardiac injury [52]. P53 directly activates Bax to permeabilize the mitochondrial membrane and initiate the apoptotic program [51]. In our study, ADSCs and the ADSC-secretome downregulated P53 and Bax levels in the injured hepatocytes, reduced caspase 3 and 9 activity, and upregulated Bcl-2 following liver ischemia-reperfusion.

The exogenous death receptor apoptosis pathway includes the tumor necrosis factor receptor (TNFR) signaling pathway, TNF-related apoptosis-inducing ligand (TRAIL) signaling pathway, and Fas ligand (Fas/Fasl) signaling pathway [29]. Studies show that all three receptors can activate caspase 8 after binding to their corresponding ligands, resulting in caspase 8 and caspase 3 cleavage causing cell apoptosis [53]. Among them, the Fas/Fasl pathway is the most detailed study of the death receptor family. Following Fas-Fasl binding, Fas undergoes trimerization, and its death domain activates caspase 8, which subsequently triggers the apoptotic cascade. Previous studies have shown that Fas and Fasl expression levels, as well as caspase 8 activity, increased rapidly after liver ischemia-reperfusion and partial hepatectomy [20]. We found that ADSCs and the ADSC-secretome significantly reduced the expression of the above factors. Consistent with our findings, Kappy found that human ADSC-derived CM protected neuroblastoma cells from apoptosis by significantly reducing Fas expression levels [54]. In addition, MSC-CM effectively reduced radiation-induced apoptosis in hepatic sinusoidal endothelial cells [41], and BMSC-CM alleviated hepatocyte apoptosis in the carbon tetrachloride-induced acute liver injury mouse model [55]. Thus, the secretome of stem cells can target both endogenous and exogenous apoptotic pathways.

At present, the route of administration of stem cells, secretome, and exosomes include systemic administration and local administration [56]. Intravenous injection is a better method of systemic administration for experimental animals, but the effective ingredients that home to the liver via the peripheral venous blood are limited, so it takes a long time to exert a therapeutic effect. The procedure of portal vein injection in large animals is complicated. In addition, there is a risk of vascular embolism, and most of the stem cells may be cleared by the liver in the early stage after portal vein injection [57]. Liver parenchymal injection for ADSC and ADSC-secretome transplantation was used in this study. However, this method of administration is also a kind of damage to the liver. Therefore, it is necessary to develop a new route of administration which should be simple to operate, widely used, safe, and effective, such as noninvasive nasal inhalation [58] or dressing in combination with a hydrogel [9]. Nevertheless, the ADSC-secretome injected into the liver parenchyma still exerts an antiapoptotic effect after HIRI combined with partial hepatectomy.

## 5. Conclusion

The ADSCs and ADSC-secretome can mitigate liver injury after HIRI combined with partial hepatectomy by blocking the endogenous and exogenous apoptotic pathways. Our findings indicate that the ADSC-secretome can inhibit hepatocyte apoptosis. The paracrine therapeutic effects of ADSCs are mediated by their secretome. Therefore, the ADSC-secretome can overcome the limitations of cell-based therapies which is a viable alternative for stem cell-based tissue repair and regeneration.

## Figures and Tables

**Table 1:** Sequences of primers used in real-time PCR.

Gene	Forward primer (5′->3′)	Reverse primer (5′->3′)
P53	CCTCACCATCATCACACTGG	TTGGCCCTTCTTGAGGAAAT
Bax	TTCAGGGTTTCATCCAGGATCG	ATCCTCTGCAGCTCCATGTTAC
Bcl-2	GAGGATTGTGGCCTTCTTTG	GCCGGTTCAGGTACTCAGTC
Fas	TGTCCGGGATCTGGGTTCTC	GGCATGGCTGACAGCAGAAT
Fasl	ACCACCACCACTCCTGCCATC	TCCCCAGCCCCAATCCAACC
*β*-Actin	TCTGGCACCACACCTTCT	TGATCTGGGTCATCTTCTCAC

## Data Availability

The datasets used and/or analyzed during this study are available from the corresponding author upon reasonable request.
